# Heterogeneous Activated B Cell Compartments Arising Early and Transiently After SARS‐CoV‐2 Vaccination

**DOI:** 10.1002/eji.70165

**Published:** 2026-03-12

**Authors:** Laura Fernandez Blanco, Lisan H. Kuijper, Laura Y.L. Kummer, Niels J.M. Verstegen, Amélie Bos, Mathieu Claireaux, Mariël C. Duurland, Tineke Jorritsma, Maurice Steenhuis, Gius Kerster, Juan J. Garcia Vallejo, Marit J. van Gils, Koos P.J. van Dam, Eileen W. Stalman, Luuk Wieske, Laura Boekel, Gertjan J. Wolbink, Sander W. Tas, Theo Rispens, Taco W. Kuijpers, Filip Eftimov, Anja ten Brinke, S. Marieke van Ham, of T2B! Immunity Against SARS‐CoV‐2 Study Group

**Affiliations:** ^1^ Sanquin Research and Landsteiner Laboratory of the Academic Medical Center University of Amsterdam Amsterdam the Netherlands; ^2^ Department of Neurology and Neurophysiology Amsterdam UMC Amsterdam the Netherlands; ^3^ Department of Medical Microbiology and Infection Prevention Amsterdam UMC, AMC Amsterdam the Netherlands; ^4^ Department of Molecular Cell Biology and Immunology Amsterdam UMC, VUmc Amsterdam the Netherlands; ^5^ Department of Clinical Neurophysiology St. Antonius Hospital Nieuwegein the Netherlands; ^6^ Amsterdam Rheumatology and Immunology Center, Reade Amsterdam the Netherlands; ^7^ Amsterdam Rheumatology and Immunology Center, Amsterdam UMC Amsterdam the Netherlands; ^8^ Department of Pediatric Immunology Rheumatology and Infectious Disease, Amsterdam UMC Amsterdam the Netherlands; ^9^ Swammerdam Institute for Life Sciences University of Amsterdam Amsterdam the Netherlands

**Keywords:** B‐cell differentiation, CD11c^+^ activated B cells, extrafollicular responses, germinal center, immunological memory, mRNA vaccination

## Abstract

In humans, the stages and dynamics of B cell development after antigen encounter remain unclear. Identifying early B cell differentiation stages could reveal biomarkers for humoral immunity and potential targets to prevent unwanted antibody responses. We characterized antigen‐specific B cell responses longitudinally after SARS‐CoV‐2 mRNA vaccination using multiparameter spectral flow cytometry. Spike‐specific IgG^+^ CD27^+^ CD71^+^ activated B cells (ActBCs), presumed to be germinal center‐derived and IgG^+^ DN2 extrafollicular B cells, dominated the early antigen‐specific B cell response, while memory B cells were the main population 6 months after vaccination. Within the IgG^+^ ActBC compartment, we delineated six novel clusters with specific contraction dynamics. Following the second vaccination, certain ActBC clusters displayed sustained expansion over time, being phenotypically similar to memory B cells, while others strongly expanded and subsequently contracted. Several of the rapidly contracting ActBC clusters expressed CD11c, a defining marker for atypical B cells, suggesting a possible extrafollicular origin of these clusters. The transient presence of heterogeneous ActBC clusters was also observed for total B cells when gated in an antigen‐independent manner. Characterization of novel ActBC clusters early after antigen encounter helps delineate and dissect the complexity of B cell differentiation, which is vital for understanding unwanted B cell responses.

## Graphical abstract

Characterization of the early antigen‐specific B cell response post‐SARS‐CoV‐2 vaccination reveals novel activated B cell clusters, showing different phenotypes and contraction dynamics. Some short‐lived activated B cells expressed both CD71 and the extrafollicular marker CD11c. These results advance our understanding of B‐cell differentiation regulation and biomarker potential.

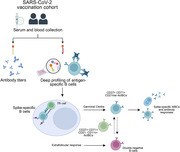

**Abbreviations**
ActBC: activated B cell
ASC: antibody‐secreting cell
DN2: double negative B cell
EF: extrafollicular
GC: germinal centers
HA: hemagglutinin
MBC: memory B cell
NCP: nucleocapsid
RBD: receptor binding domain
RSV: respiratory syncytial virus
S: spike
TT: tetanus toxoid

## 1 Introduction

Vaccination is an important strategy to battle infectious diseases by inducing long‐lasting cellular and humoral immune responses. Antibodies protect against new and recurring pathogens and are generated by antibody‐secreting cells (ASCs) induced by de novo naïve B cell differentiation or upon reactivation of memory B cells (MBCs). Both T cell‐independent and T cell‐dependent B cell differentiation routes have been identified [1, 2, 3]. Unfortunately, antibody responses can also be detrimental in the context of autoimmunity, alloimmunization, or reactivity against therapeutic biologics. Once established, long‐lived ASCs and MBCs are difficult to eradicate. Therefore, identification of ASC and MBC precursors is warranted, and knowledge of their differentiation status is much desired to design strategies to optimize vaccination responses or, conversely, prevent unwanted antibody formation.

Upon primary vaccination, a first rapid antibody response is induced. The extrafollicular (EF) pathway is a fast response that leads to the formation of short‐lived plasmablasts (PBs) and early MBCs [3, 4]. Besides this EF response, germinal centers (GC) are formed in secondary lymphoid organs where antigen‐stimulated B cells proliferate and undergo somatic hypermutation in the dark zone and repeatedly cycle to the light zone, reencounter antigen, and receive renewed T cell help [4, 5, 6]. This eventually allows for the selection of B cells with improved BCR affinity for the antigen and leads to the generation of long‐lived MBCs and ASCs. From the latter, long‐lived plasma cells are established upon migration to the bone marrow [6, 7, 8]. Using high‐output techniques, recent studies have identified diverse subphenotypes of circulatory MBCs based on different surface markers [9, 10, 11]. However, to what extent these different phenotypes share developmental trajectories or reflect different subfunctions, especially in the context of vaccination responses, requires further in‐depth research. In addition, the exact decision point and B cell stage in which bifurcation between MBC or ASC phenotype occurs remains to be elucidated. Recent studies into this subject have investigated activated B cells (ActBCs), a population of antigen‐specific B cells leaving the GC, which are phenotypically and functionally different from ASCs [11, 12, 13, 14]. Ellebedy et al. [12] identified CD71 as a marker for recently activated B cells. Lau et al. [13] defined low expression of CD21 as a marker of CD27^+^ B cells that recently emigrated from the GC and are potential PC precursors. So‐called ActBCs peak in circulation shortly after immunization or infection and drop again over time, while at the same time, circulating MBCs start to expand [9, 12, 13, 14, 15, 16]. Both classical ASC and MBC subsets have been widely studied in the context of vaccination and infection [12, 15, 17, 18]. Data are, however, lacking on the dynamics of the ActBC compartment and the further dissection of this compartment over time. Longitudinal analysis of the subsets within this compartment at different times after antigen exposure is needed to identify early B cell subsets before ASC and MBC formation has occurred, to allow their targeting in cases of unwanted B cell responses.

In recent years, the EF B cell response has acquired more attention, with a specific focus on Tbet^+^ CD11c^+^ CD21^−/lo^ cells, commonly referred to as atypical B cells, in the context of autoimmunity, chronic infections, or vaccination [14, 19, 20]. The heterogeneous nature of this population and its contribution to both normal and aberrant immune responses have led to inconsistencies in its classification and nomenclature. CD11c^+^ B cells are mainly categorized by presence or absence of CD27 expression: IgD^+^ CD27^−^ “activated‐naïve” B cells and IgD^−^ CD27^−^ ‘double‐negative 2’ B cells (DN2), identified as part of the EF response, and CD27^+^ ‘age‐associated B cells’ (ABCs), whose formation also remains unclear as they could have an EF origin or be GC‐derived and acquire CD11c upon reactivation [21]. Both populations, DN2 and ABCs, are thought to be poised for ASC differentiation, although their specific role and contribution within the B cell differentiation process remain unclear [19, 22, 23, 24, 25, 26]. Although Tbet^+^ CD11c^+^ CD21^−/lo^ B cells comprise only a small part of the total CD19^+^ B cell compartment, this population expands vastly after vaccination in humans [13, 17, 24, 27]. We recently reported DN2 and ABCs to be among the early B cell responding populations after antigen exposure and even to be the main contributors to the S (Spike)‐specific response 7 days after Severe Acute Respiratory Syndrome Coronavirus 2 (SARS‐CoV‐2) vaccination while contracting over time [24]. It remains to be established how CD27^+^ CD11c^+^ CD21^−/lo^ B cells and IgG^+^ ActBCs relate to each other and to MBCs versus PC formation.

In this study, we performed in‐depth characterization of antigen‐specific B cells to investigate the dynamics and early stages of human B cell differentiation in COVID‐19 naïve individuals after SARS‐CoV‐2 mRNA‐1273 vaccination. We studied the kinetics of S‐ and receptor binding domain (RBD)‐specific B cell populations and compared this to antigen‐specific Influenza hemagglutinin (Influenza‐HA), fusion glycoprotein from respiratory syncytial virus (RSV‐F), and Tetanus Toxoid (TT) B cell populations, which were encountered longer ago. ActBCs and EF B cells could be identified early after vaccination, followed by the establishment of resting MBCs 6 months after antigen exposure. Overall, several novel ActBC phenotypes were identified, characterized by two clear dynamics: a pronounced and significant contraction between day 7 and 6 months postvaccination, in contrast to clusters showing a sustained expansion over time, and more interdonor variation in contraction dynamics. Remarkably, a major proportion of the highly dynamic ActBC clusters expressed CD11c, which underlines that the distinction between EF and GC‐derived B cells remains to be redefined. The dynamic IgG^+^ CD11c^+^ ActBC clusters were identified in total CD19^+^ B cells without the use of antigen probes shortly after vaccination.

## 2 Methods

### 2.1 Sex as Biological Variable

Our human study examined both male and female subjects, and sex distribution was matched between our study groups.

### 2.2 Study Design and Participants

In total, 104 participants of the Target‐to‐B study cohort were used for the analysis of antigen‐specific B cells. This study cohort included healthy donors (*n* = 18), patients with rheumatoid arthritis (RA) treated with methotrexate (*n* = 16), methotrexate + TNF inhibitors (*n* = 13) or RA disease controls (*n* = 10), or inflammatory bowel disease patients (IBD) treated with TNF inhibitors (*n* = 15), purine antagonists (*n* = 15), purine antagonists + TNF inhibitors (*n* = 6), or IBD controls (*n* = 9). Further details on these cohorts are provided in previous publications [28, 29]. In the current study, we focused the analysis only on the healthy controls. Exclusion criteria were active or previous autoimmune, oncological, or hematological disease; current or previous treatment with systemic medication in the past year, pregnancy, and previous SARS‐CoV‐2 infection (as evidenced by positive anti‐RBD antibodies before first vaccination and/or self‐reported positive PCR and/or positive antinucleocapsid protein (NCP) antibodies throughout the study period). Participants were vaccinated twice between April 2021 and October 2021 with the mRNA‐1273 (Moderna) vaccine at an interval of 6 weeks. Peripheral blood was collected by venipuncture before the first vaccination, and 7 days and 6 months post second vaccination. PBMCs were cryopreserved in fetal bovine serum + 10% dimethyl sulfoxide for future use.

### 2.3 Probe Design

All recombinant, soluble proteins, including SARS‐CoV‐2 S‐2P, RBD, Influenza A hemagglutinin (H1N1pdm2009), RSV prefusion stabilized F (DS‐Cav1), were produced as described before [9]. After purification, proteins were biotinylated with a BirA500 551 biotin‐ligase reaction kit (Avidity). TT was purchased from 552 Creative Biolabs (Vcar‐Lsx003). NCP was produced as described before [30]. TT and NCP were unspecifically biotinylated using EZ‐Link Sulfo‐NHS‐LC553 Biotinylation Kit (Thermo Fisher).

### 2.4 Spectral Flow Cytometry of Antigen‐Specific B Cells

PBMCs were thawed in IMDM (Lonza) with 10% FCS (Bodinco BV). 10 × 10^6^ PBMCs were enriched for B cells using CD3 T cell depletion via the EasySep Human CD3 Positive Selection Kit II (StemCell Technologies) according to the manufacturer's instructions. To stain antigen‐specific B cells, biotinylated protein antigens SARS‐CoV‐2 S, RBD, NCP, Influenza‐HA, RSV‐F, and TT were individually multimerized with fluorochrome‐conjugated streptavidin in a 2:1 molar ratio at 4°C for 1 h. Next, 10% D‐biotin (GeneCopoeia) was added and incubated at 4°C for at least 30 min to block free sites on the streptavidin and reduce cross‐reactivity among biotinylated protein antigens. All biotinylated antigens were conjugated to two fluorochromes, except RBD, which was conjugated with a single fluorochrome and gated as part of S1 (Table S1). A 31‐color spectral cytometry panel, including the six antigen proteins, was designed to phenotype antigen‐specific B cells (Table S2). CD45RB staining was performed with the MEM‐55 clone, which targets a distinct epitope and may yield patterns differing from other clones. Enriched B cell samples were first stained using live/dead fixable blue stain kit (Invitrogen) in PBS at 4°C for 30 min. Subsequently, cells were washed twice with staining buffer (PBS supplemented with 1% BSA and 1 mM EDTA) and stained with anti‐IgG for 10 min at 4°C and immediately after with the rest of the antibody mix and antigen multimer cocktail for 30 min at 4°C. Next, cells were washed twice with PBS, fixed with 1% paraformaldehyde for 10 min at 4°C, and washed twice with staining buffer. Data were acquired on a Cytek Aurora 5L spectral cytometer using SpectroFlo software (Cytek Biosciences).

### 2.5 Spectral Flow Cytometry Data Preprocessing

After spectral unmixing (SpectroFlo v3.0.1, Cytek), FCS files were loaded into OMIQ analysis software (www.omiq.ai). Data were transformed using arcsinh with cofactors adequate for each parameter to display a Gaussian distribution of negative populations while maximizing the range of positive values. Initial gating was performed to select for single, live, and CD19^+^ DUMP^−^ (CD3, CD4, CD16, CD56) cells. PeacoQC was run to detect and remove flow cytometry anomalies in both signal acquisition and dynamic range [31]. To exclude batch effects, all data were normalized using Cytonorm for eight markers (CD19, CD20, CD24, CD38, CD45RB, HLA‐DR, IgD, and IgM) based on a reference sample, which was measured in each batch [32]. Subsequently, negative gates were set for each combination of probes, after which antigen‐specific B cells were further positively gated based on their specific combinations of fluorochrome‐conjugated streptavidin (Figure S1). For downstream analysis, data were subsampled to include only all detected antigen‐specific B cells (total Ag‐specific B cells = 450,000)

### 2.6 Dimensionality Reduction and FlowSOM Clustering

For UMAP visualization and FlowSOM clustering (xdim 16, ydim 16), input consisted of a selection of 12 markers (CD20, CD21, CD27, CD24, CD38, CD138, CD11c, CD45RB, IgA, IgD, IgM, and IgG). Antigen‐specific B cell data from 104 participants of the T2B study cohort were used. FlowSOM clusters were manually merged and annotated based on UMAP visualization, heatmap analysis of median marker expression between clusters, and manual gating of specific marker combinations (Table S3). Further downstream analysis was performed using data from the study participants included in the current paper.

### 2.7 ELISA Assays

Serum samples were collected at different time points using venipuncture and home‐based finger‐prick kits to detect the presence of RBD and NCP‐specific antibodies. An in‐house developed anti‐RBD IgG ELISA was used as described before [30, 33]. First, anti‐RBD IgG levels were measured with a quantitative ELISA and expressed as arbitrary units per milliliter (AU/mL). Second, a semiquantitative total antibody bridging ELISA to detect NCP antibodies was used to identify SARS‐CoV‐2 infection after the first vaccination [33].

### 2.8 Study Approval

This study is part of a prospective observational cohort study in the Netherlands, called the Target‐to‐B! SARS‐CoV‐2 vaccination study including healthy individuals and patients with autoimmune disease. Full study details have been previously published [34]. The study was approved by the medical ethical committee (NL74974.018.20 and EudraCT 2021‐001102‐30, local METC number: 2020_194) and registered at the Dutch Trial register (trial ID: NL8900). All participants provided written informed consent.

### 2.9 Data Availability

The Flow Cytometry Standard data generated in this study have been deposited at Zenodo (https://doi.org/10.5281/zenodo.17702364).

### 2.10 Statistical Analysis

Statistical analysis, graphs, heatmaps, and stacked bar graphs were made using RStudio (version 4.1.1) and GraphPad Prism 9.1.1. Computational analysis of data to generate heatmaps was performed using the Spectre R package [35]. Statistical significance was assessed using the Wilcoxon signed‐rank test for paired data, and *p*‐values were corrected for multiple comparisons using post hoc Bonferroni–Holm's test. *p*‐values of <0.05 were considered significant.

## 3 Results

### 3.1 Dynamics of the Antigen‐Specific B Cell Compartment Following mRNA Vaccination

**FIGURE 1:**
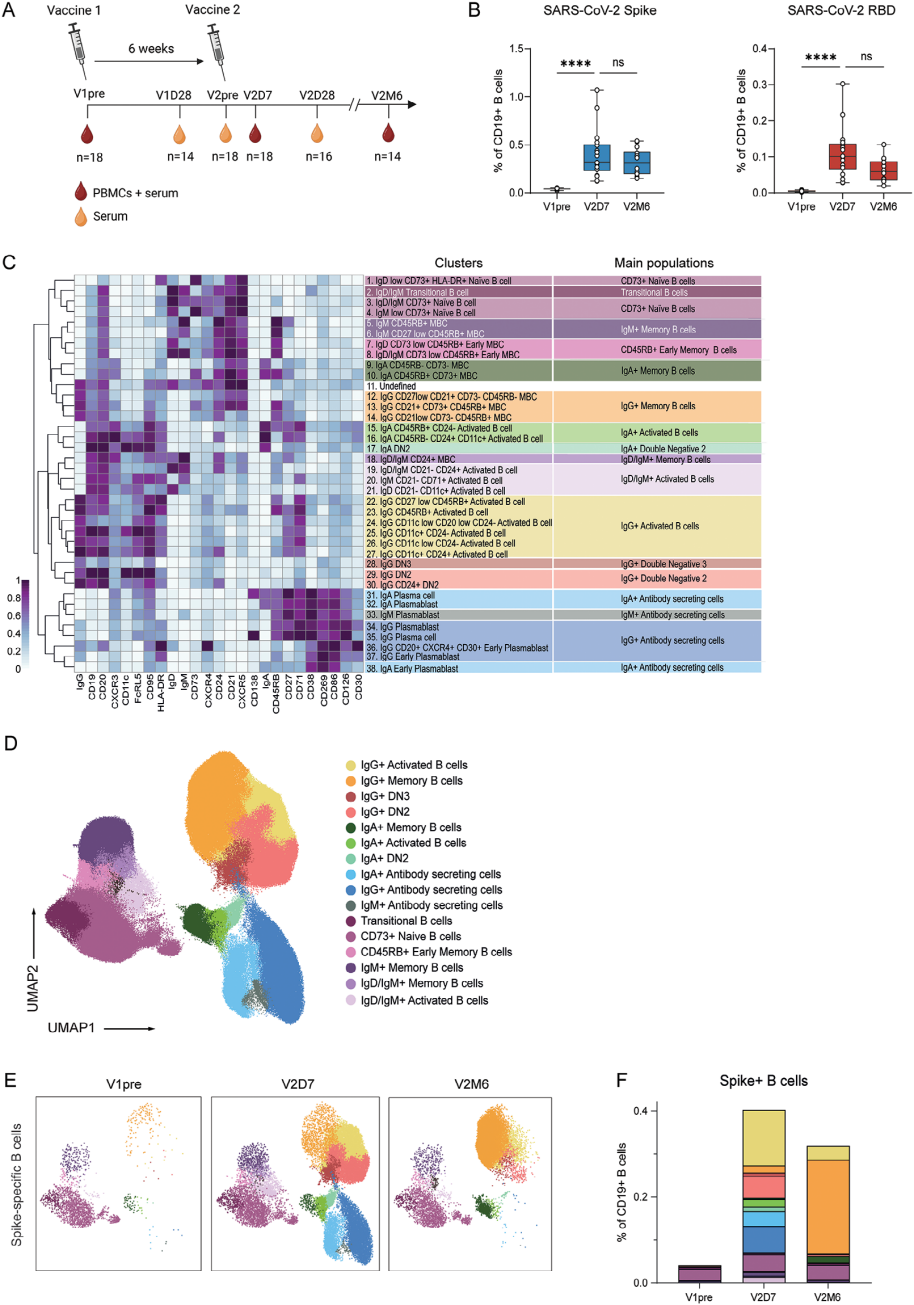
Longitudinal dynamics of the antigen‐specific B cell compartment following SARS‐CoV‐2 mRNA vaccination. **(a)** Study design and timeline with SARS‐CoV‐2 mRNA vaccination administration and peripheral blood and serum collection. (**b**) Percentage of S‐ and RBD‐ specific B cells of total CD19^+^ B cells at V1pre (*n* = 18), V2D7 (*n* = 18), and V2M6 (*n* = 14). (**c**) Heatmap of the 38 B cell clusters and 16 B cell main populations after FlowSOM clustering. (**d**) UMAP projection of all antigen‐specific B cells colored by the 16 main populations. (**e**) UMAP projection of S‐specific B cells per time point. (**f**) Stacked bar graph representing the mean proportion of S+ B cells (% of CD19^+^) for the 16 main populations per time point. Statistical significance was assessed using the Wilcoxon signed‐rank test for paired data, and *p*‐values were corrected for multiple comparison using post hoc Bonferroni–Holm's test. (*p < 0.05, **p < 0.01, ***p < 0.001, ****p < 0.0001).

To assess the dynamics of antigen‐specific B cell differentiation after recent de novo antigen exposure, from the 104 individuals in the Target‐to‐B study, a cohort of 18 SARS‐CoV‐2 mRNA vaccinated healthy individuals was selected, consisting of 10 females and 8 males with a median age of 48 years (SD = 10) (Table 1). All individuals received two mRNA‐1273 (Moderna) vaccinations and were confirmed SARS‐CoV‐2 naïve before vaccination. PBMCs were collected prior to first vaccination (V1pre, *n* = 18), seven (V2D7, *n* = 18) and 187 days (V2M6, *n* = 14) after second vaccination (Figure 1a). All individuals seroconverted after primary vaccination and remained seropositive up to 6 months post vaccination (Figure S1a; Table 1). High‐dimensional analysis was performed using a 31‐color spectral flow cytometry panel (Tables S1 and S2), including five fluorophores for combinatorial antigen labelling (S, RBD, NCP, HA, RSV‐F, TT), 21 B cell surface markers, and the four major immunoglobulin isotypes. NCP labelling and NCP antibodies were used to confirm the absence of previous SARS‐CoV‐2 infection at baseline and during the study. Of note, during the period of sampling, influenza and RSV infections were uncommon due to lockdown, and TT (DTP) revaccination was unlikely due to travel restrictions. S‐ and RBD‐specific B cells made up on average 0.4% and 0.1% of total CD19^+^ B cells, respectively, at day 7 and remained constant up to 6 months following vaccination, albeit with a declining trend for RBD (Figure 1b; Figure S1b,c). Over time, an increase in S/RBD ratio was observed (Figure S1d), indicating a shift of the SARS‐CoV‐2 specific BCR repertoire over time toward non‐RBD epitopes, in line with previously reports by us and others [36, 37].

**TABLE 1:** Subject characteristics.

	Healthy control (*n* = 18)
Age in years, mean (SD)	48 (10)
Female sex, *n* (%)	10 (55%)
Vaccine type, *n* (%)	
mRNA‐1273 (Moderna)	18 (100%)
Time between V2pre and V2post 7 days, in days, median (IQR)	7 (0)
Time between V2pre and V2post 6 months, in days, median (IQR)	187 (7)
Seroconversion after second vaccination, *n* (%)	18 (100%)
Anti‐RBD IgG titer, median (IQR)	234 (110)

To capture the phenotypic complexity and achieve the highest resolution possible of the antigen‐specific B cell response, uniform manifold approximation projection (UMAP) dimensionality reduction was used to visualize all SARS‐CoV‐2 (S, RBD) and other (TT, RSV‐F, HA) antigen‐specific CD19^+^ B cells, and subsequently FlowSOM clustering was performed on a selection of core markers (CD20, CD21, CD27, CD138, CD38, CD24, CD45RB, CD11c, IgM, IgA, IgG, IgD) on all antigen‐specific B cells of all 104 individuals (Figure S2). All further analysis was only focused on the 18 healthy individuals selected. FlowSOM consensus metaclustering was used to have enough resolution to separate less abundant B cell populations as PCs. Clustering analysis revealed 38 distinct clusters that could be manually grouped into 16 overarching B cell populations, or metaclusters, based on expression of 25 surface markers (Figure 1c,d; Figure S1e, Table S3). Among the 16 main populations, transitional B cells, CD73^+^ naïve B cells, MBCs, ActBCs, and ASCs were identified. The naïve compartment (IgD/IgM^+^ CD27^−^ CD21^+^), as visualized in the UMAP, was defined as CD73^+^ and contained a cluster of IgD^+^ IgM^lo^ B cells that could be defined as anergic B cells. Adjacent to the CD73^+^ naïve B cells in the UMAP, CD45RB^+^ early MBCs, IgD/IgM^+^ MBCs, and IgD/IgM^+^ ActBCs (CD27^−^ CD21^−^) were identified. The upper area of the UMAP depicts the IgG compartment, including IgG^+^ MBCs, IgG^+^ ActBCs (CD27^+/lo^ CD38^lo^ CD21^lo^ CD71^+^ CD95^hi^), IgG^+^ DN2 (IgD^−^ CD27^−^ CD21^−^ CD11c^+^), and IgG^+^ DN3 (IgD^−^ CD21^−^ CD27^−^ CD11c^−^) B cell populations. Additionally, MBCs, ActBCs, and DN2 populations were also observed for the IgA isotype. Lastly, the ASC compartment, annotated based on low CD19, low CD20, and high CD38 expression, represented all three BCR isotypes. Seven days after the second vaccination, the majority of the S‐ and RBD‐specific B cell response comprised of IgG^+^ ActBCs, followed by IgG^+^ and IgA^+^ ASCs and IgG^+^ DN2. After 6 months, IgG^+^ MBCs dominated the S‐ and RBD‐specific B cell response while ActBCs, DN2, and ASCs had contracted (Figure 1e,f; Figure S1f,g). Together, these data show that the ActBC, DN2, and ASC compartments dominated the antigen‐specific response 7 days after the second vaccination and contract 6 months after, while the MBC compartment is dominant 6 months after vaccination.

### 3.2 Classical Memory B Cells Can be Subphenotyped Beyond CD27 Expression Using CD21, CD73, and CD45RB

**FIGURE 2:**
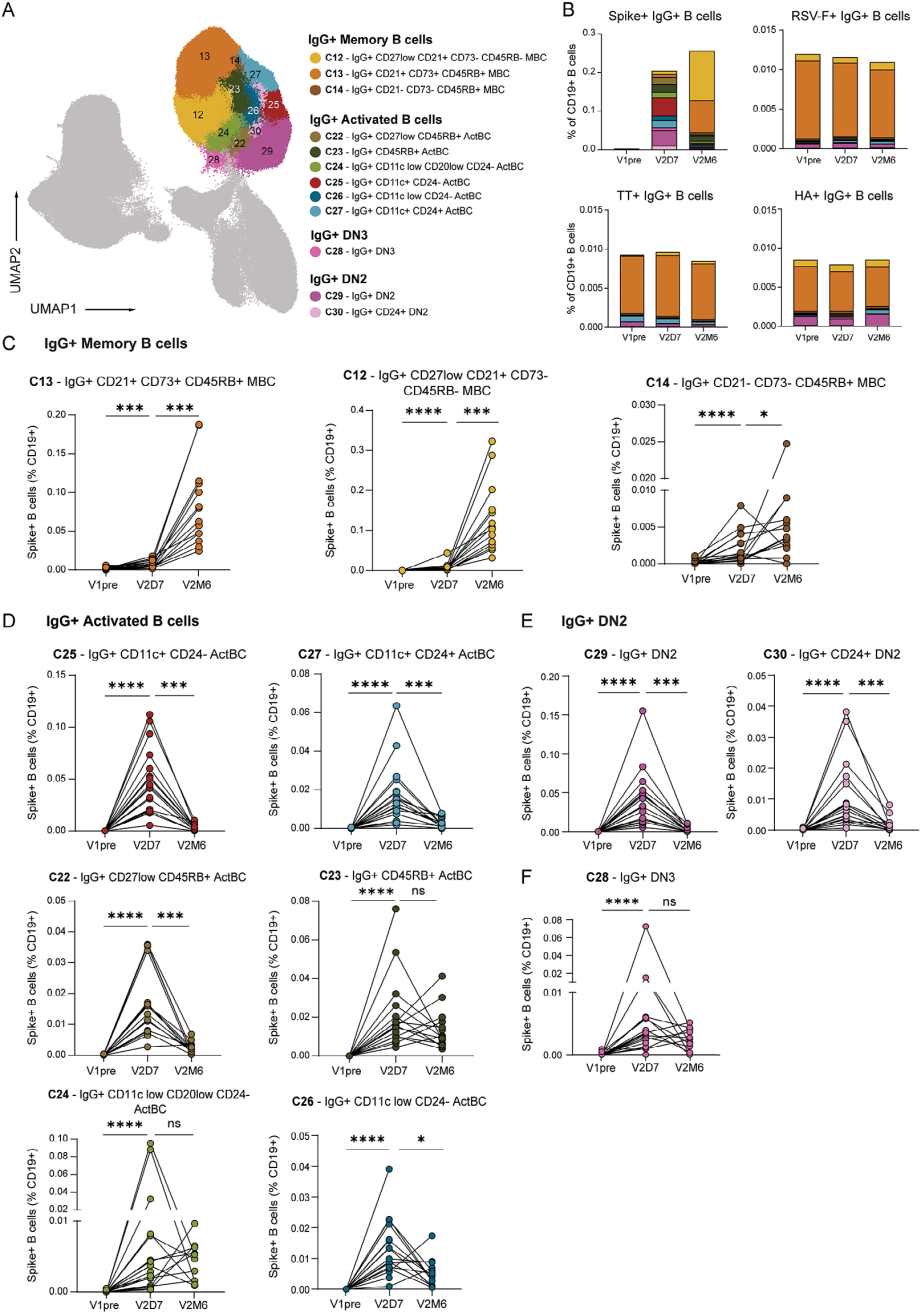
Dynamics of IgG^+^ B cell compartment after SARS‐CoV‐2 mRNA vaccination. (**a**) UMAP projection of IgG^+^ B cells, including MBCs, ActBCs, and DN, colored per cluster. (**b**) Stacked bar graph representing the mean proportion of S^+^, TT^+^, Influenza‐HA^+^, and RSV‐F^+^ IgG^+^ B cells as a percentage of CD19^+^ B cells over time. (**c**) Dynamics of IgG^+^ MBC clusters as percentage of S^+^ B cells (% of CD19^+^). (**d**) Dynamics of IgG^+^ ActBC clusters as percentage of S^+^ B cells (% of CD19^+^). (**e**) Dynamics of IgG^+^ DN2 clusters as percentage of S^+^ B cells (% of CD19^+^). (**f**) Dynamics of IgG^+^ DN3 cluster as percentage of S^+^ B cells (% of CD19^+^). V1pre (*n* = 18), V2D7 (*n* = 18) and V2M6 (*n* = 14). Statistical significance was assessed using the Wilcoxon signed‐rank test for paired data, and *p*‐values were corrected for multiple comparison using post hoc Bonferroni–Holm's test. (*p < 0.05, **p < 0.01, ***p < 0.001, ****p < 0.0001).

To further explore the dynamics and phenotypic relationships of S‐specific IgG^+^ B cells after vaccination, the IgG^+^ B cell compartment was further investigated (Figure 2a). This compartment consisted of classical MBCs, ActBCs, and DN B cells. Conventional switched MBCs are generally defined as CD27^+^ CD38^−^ CD21^+^ CD11c^−^ B cells. Recently, DN1, defined as CD27^−^ CD21^+^ CD11c^−^, has been characterized as the other circulating durable MBC population together with conventional MBCs [38]. Among switched MBCs, three different clusters (cluster 12, 13, and 14) could be distinguished. Cluster 12 and 13 dominated the S‐specific response 6 months after the second vaccination, while cluster 14 expanded less between day 7 and 6 months after vaccination (Figure 2b,c). Interestingly, cluster 12 was CD27^−^ CD21^+^ CD11c^−^. This phenotypic profile, along with its durable dynamic, is consistent with the characteristics of the DN1 population. We further analyzed these different memory phenotypes in the HA‐, TT‐, and RSV‐F‐specific B cell compartments, which have been established months to years ago. Cluster 13 dominated the HA‐, TT‐, and RSV‐F‐specific B cell compartment, while clusters 12 and 14 were only marginally present, suggesting that cluster 13 is the most long‐lived resting MBC cluster in this specific antigen context (Figures 2b,c). Cluster 13 expresses CD21, CD45RB, and a vast majority of the cells express CD73, in line with previous observations by us and others [9, 39]. CD73 is a marker for metabolic quiescence in resting MBCs, which further substantiates the long‐lived characteristic of cluster 13. Moreover, the limited representation of cluster 12 in the TT‐, HA‐, and RSV‐F‐specific compartment suggests that this DN1 population appears to be less long‐lived compared with cluster 13. Lastly, cluster 14 was CD45RB^+^ but CD73^−^ and displayed a more activated phenotype, concomitant with low CD21 expression. This phenotype, in combination with less profound expansion between day 7 and 6 months after vaccination compared with clusters 12 and 13, suggests that cluster 14 may be a more intermediate population between ActBCs and MBCs (Figure 2c).

To conclude, expression of CD73, CD21, and CD45RB defines the most sustained resting MBC compartment (cluster 13). Clusters 12 and 14 are dynamically and phenotypically related to these cells, but exhibit reduced longevity as they are much less present long after antigen exposure.

### 3.3 The IgG Activated B Cell Compartment Is a Heterogeneous Population With Diverse Dynamics Similar to Both DN B Cells and the MBC Compartment

Considering the enrichment of the S‐specific B cell compartment with IgG^+^ ActBCs and DN B cells early after vaccination [9, 14, 40], we looked deeper into the phenotype and dynamics of these compartments following vaccination (Figure 2a). The overall IgG^+^ ActBC compartment was defined based on CD27 and high CD71 expression and low expression of CD21 and CD38, whereas DN B cells were characterized by the lack of CD27, CD21, and IgD. FlowSOM clustering identified six novel different IgG^+^ ActBC clusters, based on differences in CD45RB, CD24, and CD11c expression, reflecting a high level of heterogeneity in this compartment (Figure 2a; Figure S3). Additionally, clustering revealed three different DN clusters distinguished by CD11c and CD24 expression, two defined as IgG^+^ DN2 (clusters 29 and 30) and one IgG^+^ DN3 (cluster 28) population. ActBC clusters 25, 27, and 22, although prominently present 7 days after the second vaccination, contracted almost completely 6 months after, indicative of an early, activated phenotype that is not maintained over time (Figure 2d). ActBC cluster 26 also contracted over time, but less significantly. Remarkably, clusters 25 and 27 expressed CD11c, defining an antigen‐specific ActBC population co‐expressing CD71 and CD11c (Figure S3). IgG^+^ DN2, which also expresses CD11c but lacks CD27 and CD21 expression, displayed similar dynamics over time following vaccination (Figure 2e). Interestingly, ActBC cluster 22 expressed low levels of CD27 and was located close to the DN B cells in the UMAP. On the other hand, ActBC clusters 23 and 24 did not contract significantly over time, with even expansion of these clusters in some individuals, reflecting strong interdonor variation between day 7 and month 6 after vaccination (Figure 2d). Fitting with the less contracting profile over time, cluster 23 was located close to the long‐lived MBC compartment in the UMAP (Figure 2a). In addition, cluster 23 moderately expressed CD21, indicative of an intermediate phenotype between ActBCs and MBCs, as also previously suggested for cluster 14 (Figure 2d). Interestingly, CD24, a marker recently described by our group to be expressed on activated MBCs in their trajectory to become resting MBCs, was expressed in the more dynamic cluster 27 [9]. IgG^+^ DN3 B cells, which resemble DN2 in their CD27^−^ CD21^−^ phenotype but lack CD11c expression, were less dynamic after vaccination. Collectively, these findings suggest that IgG^+^ ActBCs and DN B cells show a partial phenotypic overlap and are established early after antigen exposure, while quickly contracting in peripheral blood over time.

### 3.4 Activated B Cell Heterogeneity Is Also Evident for B Cells Expressing IgM or IgA

**FIGURE 3:**
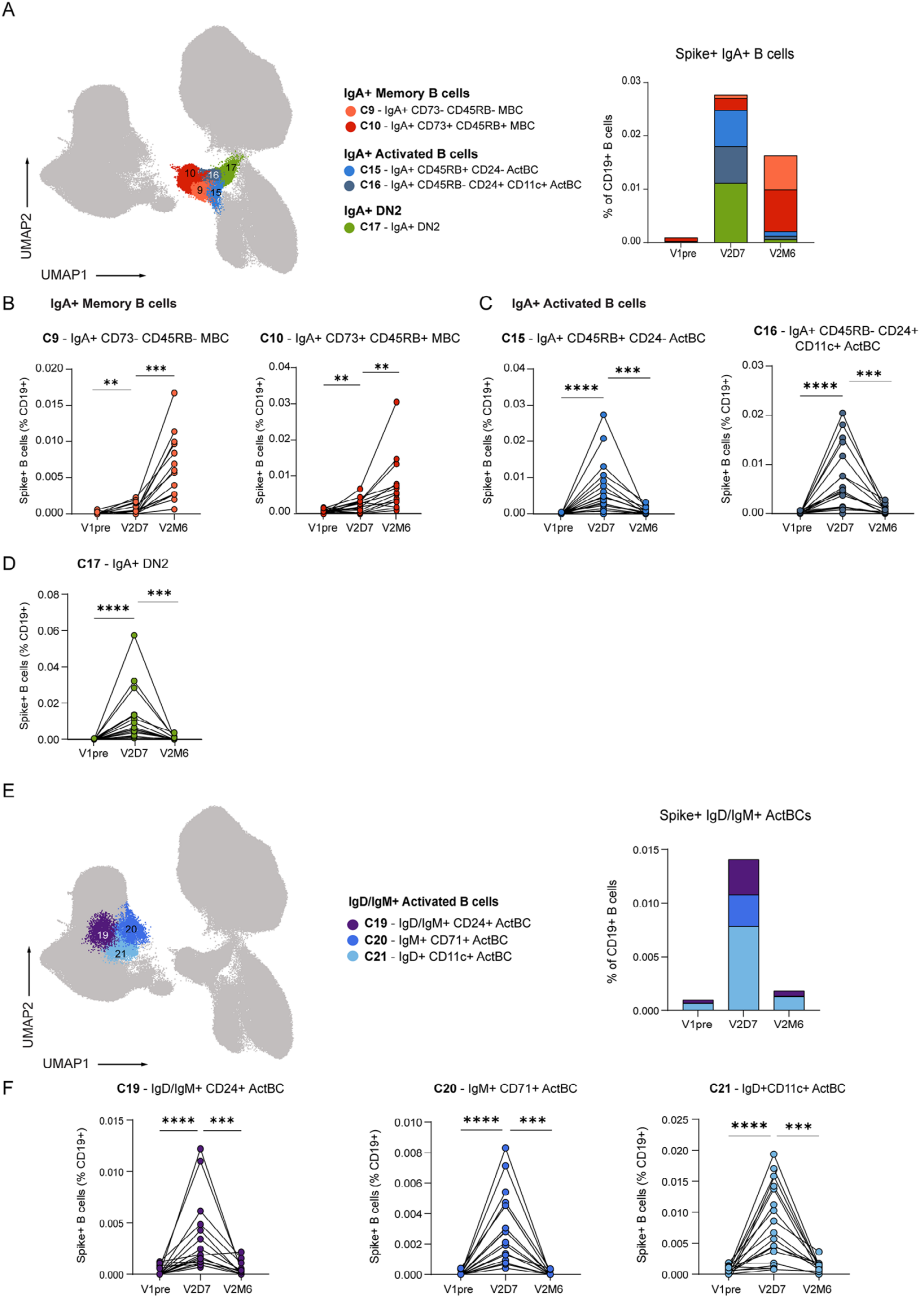
Dynamics of IgA^+^ and IgD/IgM^+^ B cell compartment after SARS‐CoV‐2 mRNA vaccination. (**a**) UMAP projection of IgA^+^ B cells, including MBCs, ActBCs, and DN2, colored per cluster. Stacked bar graph representing the mean proportion of S^+^ IgA^+^ B cells (% of CD19^+^) per cluster over time. (**b**) Dynamics of IgA^+^ MBC clusters as percentage of S^+^ B cells (% of CD19^+^). (**c**) Dynamics of IgA^+^ ActBC clusters as percentage of S^+^ B cells (% of CD19^+^). (**d**) Dynamics of IgA^+^ DN2 clusters as percentage of S^+^ B cells (% of CD19^+^). (**e**) UMAP projection of IgD/IgM^+^ ActBCs, including MBCs, ActBCs, and DN2, colored per cluster. Stacked bar graph representing the mean proportion of S^+^ IgD/IgM^+^ ActBCs (% of CD19^+^) per cluster over time. (**f**) Dynamics of IgD/IgM^+^ ActBCs clusters as percentage of S^+^ B cells (% of CD19^+^). V1pre (*n* = 18), V2D7 (*n* = 18), and V2M6 (*n* = 14). Statistical significance was assessed using the Wilcoxon signed‐rank test for paired data, and *p*‐values were corrected for multiple comparison using *post hoc* Bonferroni‐Holm's test. (*p < 0.05, **p < 0.01, ***p < 0.001, ****p < 0.0001).

To further understand the activation profile of the antigen‐specific B cell compartment after vaccination, we investigated whether the activation markers identified for the IgG^+^ B cell compartment were also indicative of IgA^+^ or IgM^+^ B cell activation. Overall, as expected, IgA^+^ B cells were less abundant than IgG^+^ B cells in the S‐specific response following vaccination, but were similar in phenotype and dynamic behavior. The IgA^+^ B cell compartment included MBCs, ActBCs, and DN2 (Figure 3a). FlowSOM clustering identified two IgA^+^ MBC clusters (clusters 9 and 10), which were mainly present later in the vaccination response. Similar to IgG^+^ MBC cluster 13, IgA^+^ MBC cluster 10 exhibited a CD21^+^ CD73^+^ CD45RB^+^ phenotype, corresponding with the long‐lasting MBC compartment (Figure 3b). Furthermore, two IgA^+^ ActBCs clusters (cluster 15 and 16) were identified. Both clusters peaked at day 7 and contracted 6 months later, but differed phenotypically in CD45RB, CD24, and CD11c expression. IgA^+^ cluster 15 was CD45RB^+^ CD24^−^ CD11c^−^, which phenotypically resembled cluster 23 in the IgG compartment. However, this cluster was short‐lived as opposed to cluster 23. IgA ^+^ cluster 16 was with CD45RB^−^ CD24^+^ CD11c^+^ expression phenotypically similar to IgG ^+^ cluster 27 (Figure 3c,d), indicating that both IgG ^+^ and IgA^+^ ActBCs can concomitantly express CD71 and CD11c.

In addition to IgA, we identified three IgD^+^ and/or IgM^+^ CD21^−^ CD27^−^ B cell clusters, located next to the CD73^+^ naïve and early MBC compartment but displaying similar dynamics to IgG^+^ and IgA^+^ ActBCs and DN2 B cells: high induction at day 7 and contraction at 6 months. The three clusters additionally expressed CD24 (cluster 19), CD71 (cluster 20), or CD11c (cluster 21) (Figure 3e,f). Cluster 21 (IgD^+^ CD11c^+^ ActBCs), previously referred to as ‘activated‐naïve’ B cells [24], was the most abundant of all three clusters.

Together, these results demonstrate that the IgA and IgD/IgM activated compartments are heterogeneous, as seen for IgG. Also, in IgD/IgM^+^ B cells, the absence of CD21 and the expression of CD71, CD11c, or CD24 are indicators of B cell differentiation states that arise early after antigen encounter.

### 3.5 Preplasmablasts Show Phenotypic Similarity With ActBCs and DN3 B Cells

**FIGURE 4:**
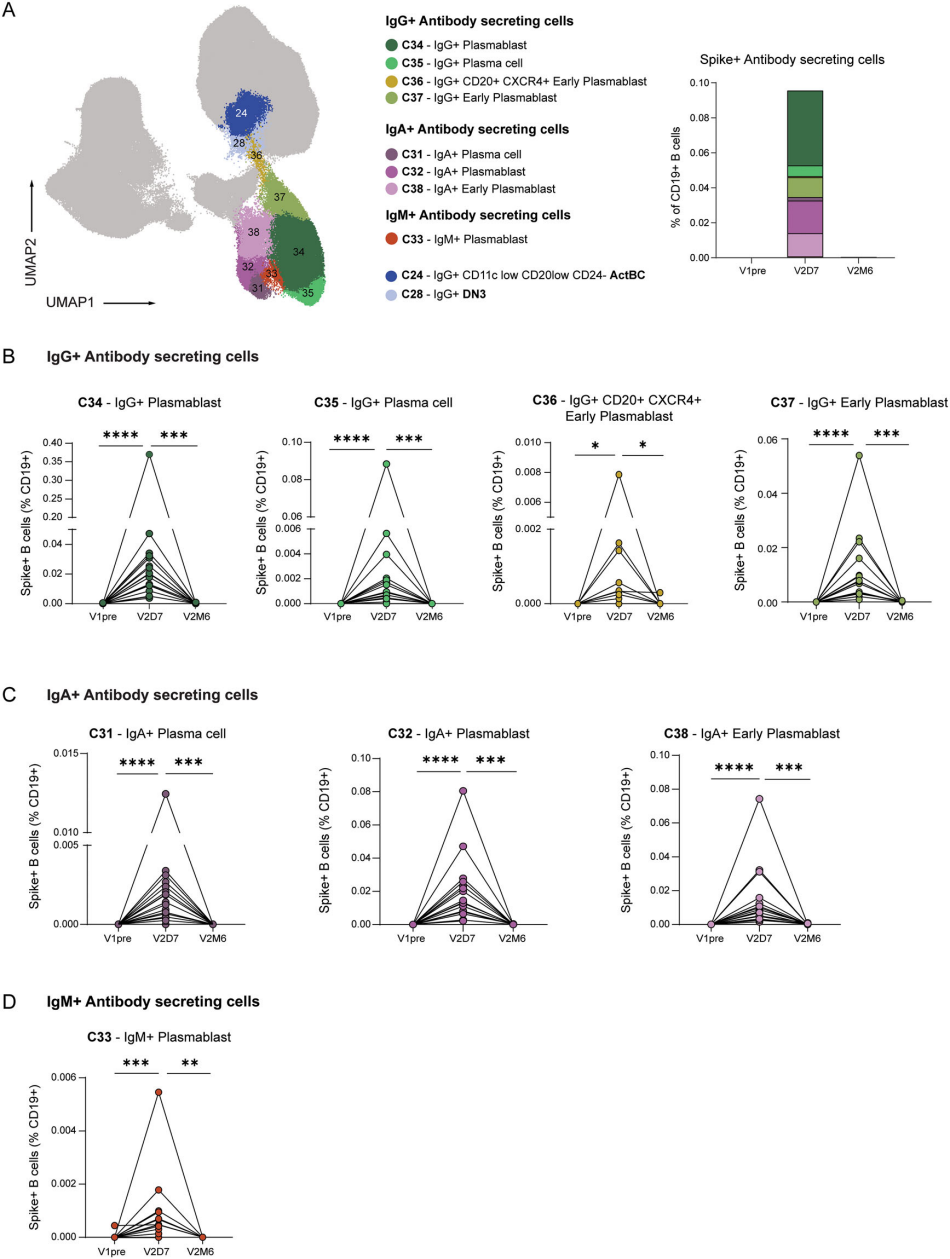
Dynamics of the ASC compartment after SARS‐CoV‐2 mRNA vaccination. (**a**) UMAP projection of IgG^+^, IgA^+^, and IgM^+^ ASC clusters. Clusters 24 and 28 are also depicted in the UMAP. Stacked bar graph representing the mean proportion of S^+^ ASCs (% of CD19^+^) per cluster over time. (**b**) Dynamics of IgG^+^ ASC clusters. (**c**) Dynamics of IgA^+^ ASC clusters. (**d**) Dynamics of IgM^+^ ASC cluster. V1pre (*n* = 18), V2D7 (*n* = 18), and V2M6 (*n* = 14). Statistical significance was assessed using the Wilcoxon signed‐rank test for paired data, and *p*‐values were corrected for multiple comparison using post hoc Bonferroni–Holm's test. (*p < 0.05, **p < 0.01, ***p < 0.001, ****p < 0.0001).

Given the fragility of ASCs and since frozen cells were used, it is likely that we underestimated the frequency of the ASC compartment; still we were able to identify eight different clusters [17, 39]. The IgG^+^ ASCs consisted of PCs (CD27^hi^ CD38^hi^ CD138^+^), PBs (CD27^hi^ CD38^hi^), and early PBs (CD27^lo^ CD38^+^) clusters, with IgG^+^ PB as the most abundant cluster. As described before, all clusters demonstrated similar dynamics of expansion early after vaccination and contraction 6 months after (Figure 4). Interestingly, a minor IgG^+^ CD20^+/int^ CXCR4^+^ CD30^+^ cluster 36 was identified. Similar to pre‐PB clusters, cluster 36 was CD38^+^ CD27^lo^ and had high CD269 expression, but was unique in high CD30 and intermediate CD20 expression (Figure 4a,b). Furthermore, cluster 36 was located in the UMAP next to IgG^+^ ActBC cluster 24 and IgG^+^ DN3 B cells (cluster 28) (Figure 4a). Both clusters showed less CD20 expression than other B cell populations, suggesting that these clusters might be related to pre‐PB cells.

### 3.6 The Early and Transient Activated B Cell Subpopulations Can Also be Identified by Manual Gating After Antigen Encounter

**FIGURE 5:**
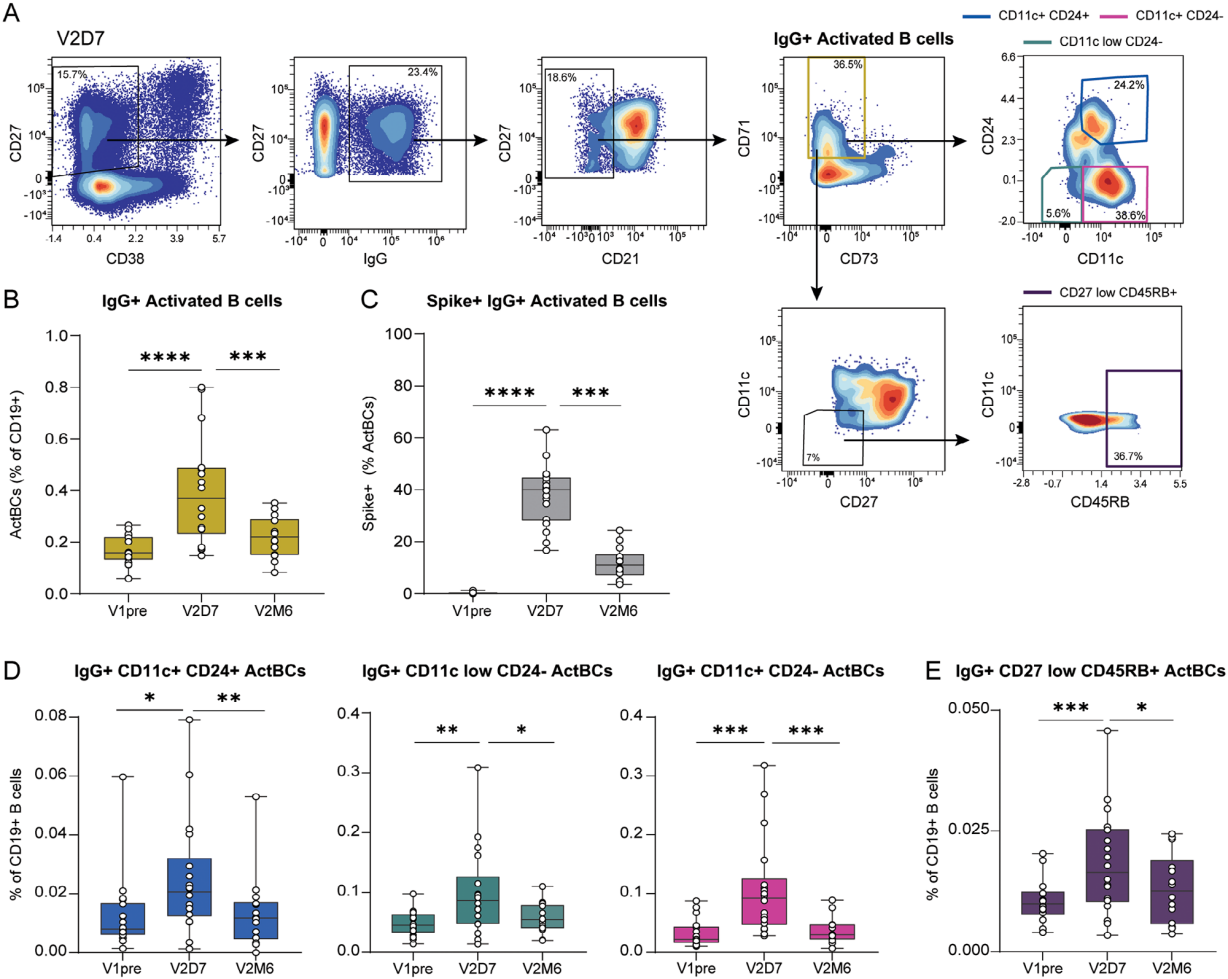
Gating strategy of early and transient activated B cell subpopulations after antigen encounter. (**a**) Representative gating strategy to detect IgG^+^ ActBCs in total CD19^+^ B cells from healthy donor PBMCs at V2D7, and further CD24, CD11c, CD27, and CD45RB gating strategy to detect early and transient B cell subpopulations. (**b**) Proportion of IgG^+^ ActBCs as a percentage of CD19^+^ B cells. (**c**) Proportion of Spike^+^ B cells as a percentage of IgG^+^ ActBCs. (**d**) Proportion of IgG^+^ CD11c^+^ CD24^+^, CD11c^+^ CD24^−^, CD11c^lo^ CD24^−^ ActBCs as a percentage of CD19^+^ B cells. (**e**) Proportion of IgG^+^ CD27^lo^ CD45RB^+^ ActBCs as a percentage of CD19^+^ B cells. V1pre (*n* = 18), V2D7 (*n* = 18), and V2M6 (*n* = 14). Statistical significance was assessed using the Wilcoxon signed‐rank test for paired data, and *p*‐values were corrected for multiple comparison using post hoc Bonferroni–Holm's test. (*p < 0.05, **p < 0.01, ***p < 0.001, ****p < 0.0001).

Unsupervised clustering allowed us to perform in‐depth characterization of the ActBC compartment and provided indications of early activated B cell populations that quickly respond to antigen encounter. For many B‐cell‐mediated diseases, the (auto)antigen is unknown, but tracking the active B‐cell response is of great importance to assess disease activity, as a marker of relapse and treatment efficacy. We have previously shown by manual gating that around 20% of S‐specific B cells were CD27^+^ CD11c^+^ 7 days after SARS‐CoV‐2 vaccination [24]. Therefore, we investigated whether the unique phenotypic profiles of highly dynamic ActBC populations could also be employed to track active B cell responses in total CD19^+^ B cells, without the need for antigen labelling. We focused on the transient ActBC clusters 22, 25, 26, and 27 together to track the early B cell response after vaccination. Total ActBCs were manually gated from bulk CD19^+^ B cells by selecting for IgG^+^ CD27^+/lo^ CD38^lo^ CD21^−^ CD71^+^ B cells (Figure 5a; Figure S4). We were able to identify a significant expansion of IgG^+^ ActBCs at day 7 after the second vaccination (Figure 5b). Approximately 38% of the IgG^+^ ActBC expansion 7 days after the second vaccination was S‐specific compared with 0.2% at baseline and 11% 6 months after vaccination, indeed showing that shortly after exposure, the ActBC compartment is enriched for B cells specific for the recently encountered antigen (Figure 5c). To further explore this compartment, we gated on CD11c^+^ CD24^+^, CD11c^+^ CD24^−^, and CD11c^lo^ CD24^−^ to be able to capture clusters 27, 25, and 26 (Figure 5a). We found that these ActBC subpopulations were expanded at day 7 after the second vaccination and also significantly reduced 6 months after, indicating that this combination of markers can be used to detect transient activated B cell responses without the use of antigen‐specific probes (Figure 5d). Cluster 22 also showed a transient dynamic after vaccination, so we then gated on CD27^lo^ CD11c^−^ CD45RB^+^ ActBCs and also confirmed this dynamic of early expansion and significant contraction between day 7 and 6 months after vaccination (Figures 5a,e).

Overall, our findings reveal deeper insights into the transient phenotypes of the ActBC compartment. Within this compartment, we identified, in total CD19^+^ B cells, four distinct clusters that expand early and rapidly contract over time based on the marker combination of CD11c, CD24, CD45RB, and CD27. These clusters represent the most promising candidates for tracking autoreactive B cells, highlighting their fundamental role in early immune responses.

## 4 Discussion

Although recent advances have been made in characterizing the phenotypic diversity of the different B cell stages following antigen exposure [9, 11, 17, 24, 39, 40], better insights into the different populations that occur early after antigen exposure in the immune response will support further understanding, monitoring, and possible intervention in the B cell differentiation response. In this study, we have built a comprehensive and dynamic atlas of Bcell populations present at different stages during an active immune response by performing deep immune profiling of antigen‐specific B cells in a longitudinal cohort study following SARS‐CoV‐2 vaccination in healthy individuals. We have demonstrated a higher level of complexity of the antigen‐specific B cell responses than was previously acknowledged.

Classical CD27^+^ MBCs are the B cells generically used to measure the size of the memory B cell compartment after antigen‐experience. They are generally defined as a homogenous durable population of antigen‐experienced B cells. Using high‐throughput approaches, recent advances have been made in defining different subphenotypes of circulatory MBCs [9, 10, 11]. DN1 B cells have been recently described as a durable MBC population, defined as class‐switched CD27^−^ CD21^+^. Unlike conventional MBCs, they are believed to be nongerminal‐center‐derived [38]. Characterizing the different MBC phenotypes and their longevity holds great value, especially for optimizing vaccination responses and for detecting undesired MBC responses as in the context of autoimmunity. Both S‐specific IgG^+^ and IgA^+^ CD21^+^ CD73^+^ CD45RB^+^ MBCs were present most prominently 6 months following vaccination. In addition, this CD21^+^ CD73^+^ CD45RB^+^ MBC cluster was the most dominant IgG^+^ B cell cluster observed for HA‐, TT‐, and RSV‐F‐specific antigens, which were encountered longer ago in our cohort, defining the truly long‐lived resting MBC compartment [9, 39]. Our data also showed CD27^lo^ CD21^+^ CD73^−^ CD45RB^−^ MBCs to be strongly expanded 6 months after vaccination, in line with the definition of DN1 and their longevity. Interestingly, they did not contribute to the long‐lasting memory compartment of previously encountered antigens. This cluster has also been observed by other studies 1 year after Influenza and SARS‐CoV‐2 vaccination [11, 41], and may reflect an extrafollicular MBC population that contributes to maintaining immunological breath in response to antigens with repeated exposure, with the possibility to reenter the GC. Lastly, the smallest observed MBC population (CD21^−^ CD73^−^ CD45RB^+^, cluster 14) might resemble the naïve‐proximal MBC population described by McGrath et al. [10], given its expression of CD45RB and absence of CD73. Together, our data and that of others, show that the CD21^+^ CD73^+^ CD45RB^+^ marker combination seems to be the phenotype that distinguishes long‐lived IgG^+^ MBCs.

Upon identification of the early B cell compartment, we found that IgG^+^ ActBCs dominated the S‐specific response, together with ASCs and DN2 (CD27^−^ CD21^−^ CD11c^+^) at day 7 after vaccination, consistent with findings reported by others [14, 20, 40, 42]. ActBCs have been identified as GC‐experienced B cells, which are present early after antigen exposure [9, 12, 13, 14]. Inconsistencies between different studies in defining and naming ActBCs have made comparisons between populations challenging. ActBCs are generally defined as CD27^+^ CD21^−^ B cells [9, 12, 13, 14], while others refer to them as activated MBCs [15, 40, 41, 43]. Here, we defined the ActBC compartment as CD27^+/lo^ CD38^lo^ CD21^lo^ CD71^+^ CD95^hi^ B cells, combining the absence of CD21 with the expression of CD71 and CD95 as also employed by others [9, 12, 14]. From this definition and from our clustering analysis, CD71 was the best discriminative marker to capture the ActBC compartment. ASCs also express CD71, but this compartment can be discriminated by being CD19^−/lo^ and low in BCR signal, as observed for IgG and IgA expression. Notably, we found that the ActBC compartment was CD38^lo^ while others define it as CD38^+^ [14, 40]. This likely can be explained by the fact that these studies looked at the relative CD38 expression within the B cells, excluding the CD38^hi^ ASCs. In our study, all CD38^hi^ CD71^+^ cells were confined to the ASC compartment.

Deep profiling of the IgG^+^ ActBC compartment allowed us to subdivide it into six clusters based on differential expression of certain markers and distinct dynamics, together providing a higher resolution of the ActBC compartment. The six clusters could dynamically be divided into two groups, with both groups arising early after antigen exposure, but one group contracting faster than the other in the long term. Clusters 23 and 24 arose early after antigen encounter, but did not contract in all individuals over time. It is tempting to speculate that the prolonged presence of these clusters 6 months after vaccination may suggest that they were induced during a longer period in GC reactions, in line with the persistent GC after mRNA vaccination and the suggested GC‐experienced nature of IgG^+^ ActBCs in general [12, 13, 37, 44].

The early arising ActBC clusters 22, 25, 26, and 27 contracted between day 7 and month 6 after vaccination. One of our most striking findings was the co‐expression of CD71 and CD11c in the IgG^+^ ActBC clusters 25 and 27. Indeed, our previous findings revealed by manual gating that around 20% of S‐specific B cells were CD11c‐expressing CD27^+^ CD38^−^ B cells, referred to as age‐associated (atypical) B cells, 7 days after SARS‐CoV‐2 vaccination, while CD71 expression was not regarded [24]. IgG^+^ DN2, which lack CD27 and CD21 expression but express CD11c, also arose early and contracted to prevaccination levels 6 months after vaccination. DN2 are thought to be part of the EF B cell response and have been extensively studied in the context of chronic infections and autoimmunity [19, 23]. Current unsupervised analysis and previous manual gating [24] indicate that, besides ActBCs, DN2 cells are also a relevant antigen‐specific compartment of the active vaccination response. The similar dynamics of the CD11c^+^ ActBCs with DN2 and the finding that they contract more in comparison to the other ActBC (clusters 23, 24) populations, may indicate that CD11c^+^ ActBCs and DN2 could share an EF origin. However, the similar dynamics in contraction with the CD11c^−^ ActBC cluster 22 does not support that hypothesis. This uncertainty is underscored by a recent study showing plasticity between human CD21^+^ CD27^+^ resting and CD21^−^ CD27^+^ activated MBCs and DN2 (CD21^−^ CD27^−^) during SARS‐CoV‐2 recall response, also revealing clonality relationships between the different MBC subsets and DN2 [15]. All in all, the position of CD11c^+^ CD71^+^ ActBC clusters in EF‐ or GC‐derived B cell differentiation remains to be elucidated by further research. Future analysis of the level of somatic hypermutation and clonal relationships between these clusters and the long‐lived MBCs are needed to define how these populations relate to each other and which clusters are precursor stages of GC‐derived long‐lived MBC.

In addition to investigating the relationships between ActBCs, long‐lived MBCs, and EF B cells, several studies have aimed to address whether certain subsets of ActBCs contribute to the resting MBC pool or are poised for transition to ASC lineage [12, 13, 40]. In our data, in the ASC compartment, cluster 36 might represent a pre‐ASC B cell stage, as it still expressed CD20 and was located in a phenotypic continuum between IgG^+^ ActBCs and IgG^+^ ASCs in the UMAP. In addition, this cluster was high in CD30 expression, in line with recent assignments of CD30 expression to a pre‐PB GC‐derived population [16, 45, 46]. It is important to highlight that cluster 36 is in a phenotypic continuum with the DN3 cluster and the IgG^+^ ActBC cluster 24. As these clusters have lower expression of CD20 compared with the other ActBCs, they may represent good candidates predisposed for ASC differentiation. Further research on isolated subsets involving scRNAseq and analyses of clonal evolution over time is needed to identify the decision point between MBCs and ASCs.

Finally, our data demonstrate that an increase in transient IgG^+^ ActBC clusters early after antigen exposure can be detected in the total CD19^+^ B cell compartment by gating on IgG^+^ CD27^+/lo^ CD38^lo^ CD21^−^ CD71^+^ B cells in combination with CD11c, CD24, and CD45RB. Detection of early activated B cells, correlating with recent antigen exposure, without the use of antigen‐specific probes, offers the potential to monitor recently activated B cells with unknown antigen specificity. For many detrimental B cell responses, for instance, in the context of autoimmunity, the autoantigens that drive the B cell response are unknown. This hampers the investigation and identification of the autoreactive B cell compartments. Consequently, monitoring of the autoreactive B cell compartment before therapy and the effects of therapy on this compartment cannot be executed. As our gating strategy now allows the identification of transient IgG^+^ ActBC clusters in total B cells, it now become feasible to assess if these clusters can be used as a proxy for autoreactive B cells having recently encountered auto‐antigen. This is the case, for instance, with autoimmune diseases of acute onset, such as myasthenia gravis or encephalomyelitis. Next steps would be to assess if effective therapy would diminish the size of this compartment and, if so, if the specific transient IgG^+^ ActBC clusters would also have the potential to identify patients prone to relapses before the onset of actual clinical symptoms.

To conclude, this study provides a detailed and dynamic map of the antigen‐specific B cell compartment after antigen exposure. The revelation of different ActBC clusters with different phenotypes and contraction dynamics brings us closer to understanding B cell differentiation trajectories toward either MBCs or ASCs. The possibility of tracking an active immune response without the use of antigen labeling offers new opportunities to explore early B cell differentiation in response to unknown antigens, as in the context of autoimmune diseases. This information lays the framework for future research on identifying factors regulating human B cell differentiation. Further investigation of the ActBC compartment may allow identification of long‐lived ASC or MBC precursors that could be potentially targeted in the context of unwanted antibody formation or for improving vaccination strategies against existing and emerging pathogens.

### 4.1 T2B! Immunity Against SARS‐CoV‐2 Study Group

Renée CF van Allaart, Adája E Baars, Marcel W Bekkenk, Frederike J Bemelman, Angela L Bosma, Bo Broens, Esther Brusse, Matthias H Busch, Olvi Cristianawati, Pieter A van Doorn, George Elias, Cécile ACM van Els, H Stephan Goedee, Dirk Jan Hijnen, Marc L Hilhorst, Barbara Horváth, Papay BP Jallah, Mark Löwenberg, Elham S Mirfazeli, Annelie H Musters, Jim BD Keijser, Sofie Keijzer, Zoé van Kempen, Joep Killestein, Karina de Leeuw, Anneke J van der Kooi, Lotte van Ouwerkerk, Pieter van Paassen, Agner R Parra Sanchez, W Ludo van der Pol, Nicoline F Post, Joost Raaphorst, Annabel M Ruiter, Abraham Rutgers, Carolien E van de Sandt, Corine RG Schreurs, Phyllis I Spuls, R Bart Takkenberg, YK Onno Teng, Yosta Vegting, Jan JGM Verschuuren, Adriaan G Volkers, Alexandre E Voskuyl, Jelle de Wit, Diane van der Woude, and Koos AH Zwinderman.

## Author Contributions

**Anja ten Brinke** and **S. Marieke van Ham** led the study. **S. Marieke van Ham** is the guarantor. **S. Marieke van Ham** and **Taco W. Kuijpers** supervised the study. **Filip Eftimov**, **Taco W. Kuijpers,** and **S. Marieke van Ham** conceptualized the study. **S. Marieke van Ham**, **Anja ten Brinke**, **Laura Fernandez Blanco**, **Lisan H. Kuijper**, **Amélie Bos**, **Niels J.M. Verstegen**, **Mathieu Claireaux**, **Marit J. van Gils**, **Juan J. Garcia Vallejo**, **Theo Rispens,** and **Maurice Steenhuis** designed the methodology. **Lisan H. Kuijper**, **Laura Y.L. Kummer**, **Amélie Bos**, **Laura Fernandez Blanco**, **Niels J.M. Verstegen**, **Mariël C. Duurland**, **Tineke Jorritsma,** and **Gius Kerster** performed the experiments. **Laura Fernandez Blanco**, **Lisan H. Kuijper**, **Laura Y.L. Kummer**, **S. Marieke van Ham,** and **Anja ten Brinke** verified the overall replication/reproducibility of the research output. **Laura Fernandez Blanco**, **Lisan H. Kuijper**, **Laura Y.L. Kummer**, **Amélie Bos,** and **Niels J.M. Verstegen** analyzed the data. **Laura Y.L. Kummer**, **Koos P.J. van Dam**, **Eileen W. Stalman**, **Laura Boekel**, **Gertjan J. Wolbink,** and **Sander W. Tas** provided study materials. **Laura Y.L. Kummer**, **Koos P.J. van Dam**, **Eileen W. Stalman,** and **Luuk Wieske** managed the research data. **Laura Y.L. Kummer**, **Koos P.J. van Dam**, **Eileen W. Stalman**, **Luuk Wieske**, **Theo Rispens**, **Taco W. Kuijpers**, **Filip Eftimov**, **S. Marieke van Ham,** and **Anja ten Brinke** managed and coordinated the research activity and planning. **Taco W. Kuijpers**, **Filip Eftimov,** and **S. Marieke van Ham** acquired the financial support for the project leading to this publication. **Laura Y.L. Kummer**, **Lisan H. Kuijper**, **Mathieu Claireaux,** and **Niels J.M. Verstegen** designed and implemented computer codes. **Laura Fernandez Blanco** visualized the work. **Laura Fernandez Blanco**, **S. Marieke van Ham,** and **Anja ten Brinke** wrote the original draft. All authors reviewed and approved the manuscript.

## Funding

All funding sources have been included in the Acknowledgments.

## Ethics Statement

This study was approved by the medical ethical committee (NL74974.018.20 and EudraCT 2021‐001102‐30, local METC number: 2020_194). Participants gave informed consent to participate in the study before taking part.

## Conflicts of Interest

Filip Eftimov, Gertjan J. Wolbink, S. Marieke van Ham, and Taco W. Kuijpers report (governmental) grants from ZonMw to study immune response after SARS‐CoV‐2 vaccination in autoimmune diseases. Filip Eftimov also reports grants from Prinses Beatrix Spierfonds, CSL Behring, Kedrion, Terumo BCT, Grifols, Takeda Pharmaceutical Company, and GBS‐CIDP Foundation; consulting fees from UCB Pharma and CSl Behring; honoraria from Grifols. The remaining authors declare no conflicts of interest.

## Supporting information




**Supporting File**: eji70165‐sup‐0001‐SuppMat.pdf.

## Data Availability

Data sharing policy does not apply to this article. No data has been generated.
