# Supplementary material for: Heterogeneous Activated B Cell Compartments Arising Early and Transiently After SARS‐CoV‐2 Vaccination
Source: Eur J Immunol. 2026 Mar 12;56(3):e70165. doi: 10.1002/eji.70165 (PMC12981206; doi:10.1002/eji.70165)
Supplement: Supplementary file 1 — Supporting File: eji70165‐sup‐0001‐SuppMat.pdf. [file EJI-56-e70165-s001.pdf]

Supplementary Information

Heterogenous activated B cell compartments arising early and transiently after antigen exposure

Authors:

Laura Fernandez Blanco[1,2*‡], Lisan H Kuijper[1*], Laura YL Kummer[1,2*], Niels JM Verstegen[1], Amélie Bos[1], Mathieu Claireaux[3], Mariël C Duurland[1], Tineke Jorritsma[1], Maurice Steenhuis[1], Gius Kerster[3], Juan J Garcia Vallejo[4], Marit J van Gils[3], Koos PJ van Dam[2], Eileen W Stalman[2], Luuk Wieske[5], Laura Boekel[6], Gertjan J Wolbink[6], Sander W Tas[7], Theo Rispens[1], Taco W Kuijpers[8], Filip Eftimov[2], Anja ten Brinke[1#], S Marieke van Ham[1,9#,‡]; On behalf of T2B! Immunity against SARS-CoV-2 study group

Affiliations:

[1]Sanquin Research and Landsteiner Laboratory of the Academic Medical Center, University of Amsterdam, Amsterdam, the Netherlands

[2]Department of Neurology and Neurophysiology, Amsterdam UMC, location AMC, Amsterdam, the Netherlands

[3]Department of Medical Microbiology and Infection Prevention, Amsterdam UMC, location AMC, Amsterdam, the Netherlands

[4]Department of Molecular Cell Biology and Immunology, Amsterdam UMC, location VUmc, Amsterdam, the Netherlands

[5]Department of Clinical Neurophysiology, St. Antonius Hospital, Nieuwegein, the Netherlands

[6]Amsterdam Rheumatology and Immunology Center, location Reade, Amsterdam, the Netherlands

[7]Amsterdam Rheumatology and Immunology Center, Amsterdam UMC, Amsterdam, the Netherlands

[8]Department of Pediatric Immunology, Rheumatology and Infectious Disease, Amsterdam UMC, Amsterdam, the Netherlands

[9]Swammerdam Institute for Life Sciences, University of Amsterdam, Amsterdam, the Netherlands

*Shared first authors

#Shared last authors

‡Corresponding author: Laura Fernandez Blanco, Sanquin Research, Plesmanlaan 125, 1066 CX, Amsterdam, The Netherlands. Email: l.fernandezblanco@sanquin.nl; Prof. Dr. S.M. van Ham, Sanquin Research, Plesmanlaan 125, 1066 CX, Amsterdam, The Netherlands email: m.vanham@sanquin.nl

Supplementary Figure 1

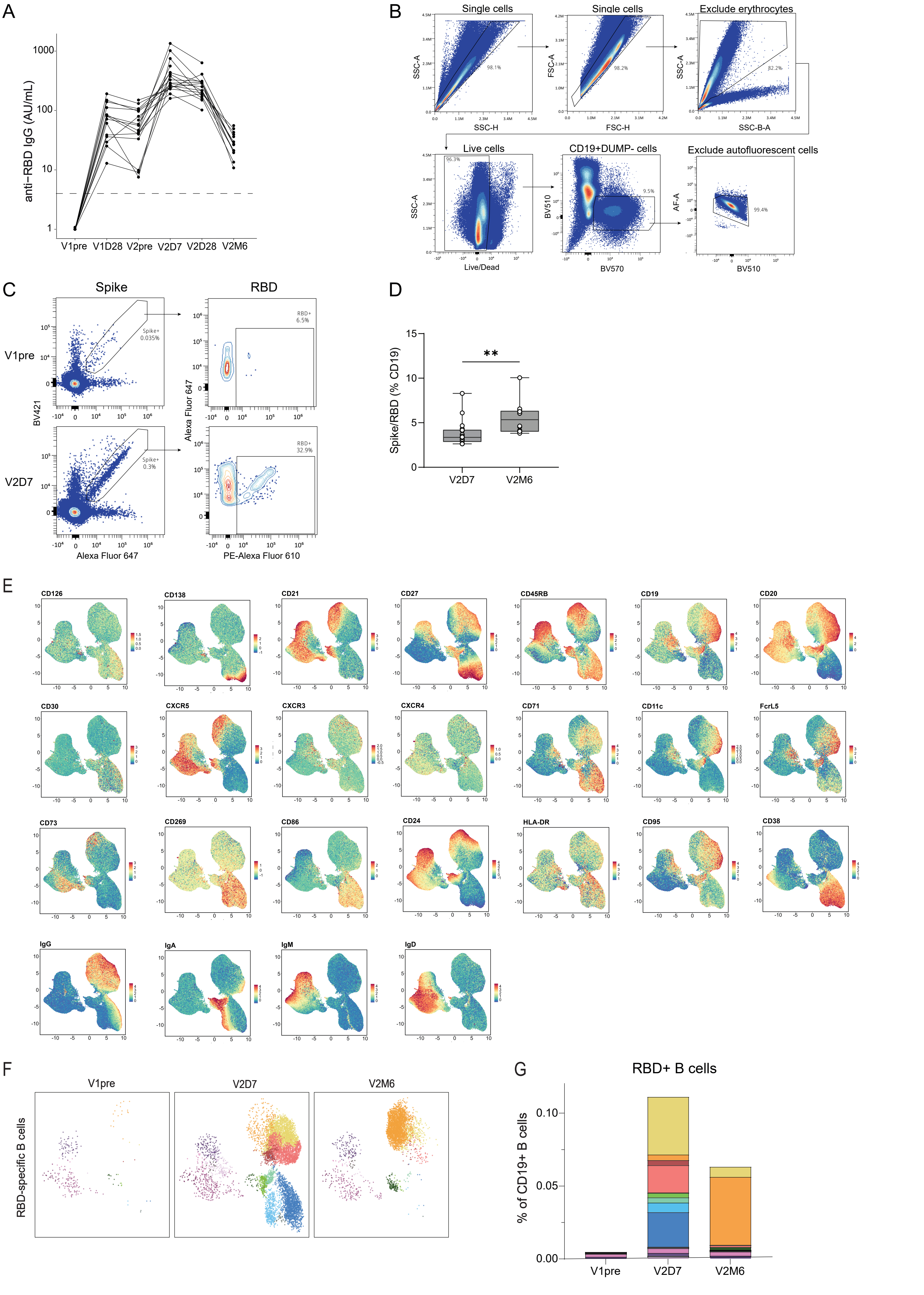

Supplementary fig. 1. SARS-CoV-2-specific antibody and B cell responses.

a. Anti-RBD IgG levels for all 18 individuals at V1pre, V1D28, V2pre, V2D7, V2D28, V2M6. **b.** Gating strategy for the detection of CD19+ B cells. **c.** Gating strategy for the detection of S-specific B cells using a combinatorial probe staining. S-specific B cells are detected as double positive for the binding of the same antigen combined with two different fluorophores. RBD-specific B cells are detected from S-specific B cells. **d.** Percentage of S/RBD-ratio of total CD19+ B cells at V2D7 (n=18) and V2M6 (n=14) **e.** UMAP representation showing normalized expression of all markers in all antigen-specific B cells. **f.** UMAP projection of RBD-specific B cells per time point. **g.** Stacked bar graph representing the mean proportion of RBD+ B cells (% of CD19+) for the 16 main populations per time point. Statistical significance was assessed using Wilcoxon signed-rank test for paired data and p-values were corrected for multiple comparison using *post hoc* Bonferroni-Holm's test. (*$P < 0.05$, **$P < 0.01$, ***$P < 0.001$, ****$P < 0.0001$).

Supplementary Figure 2

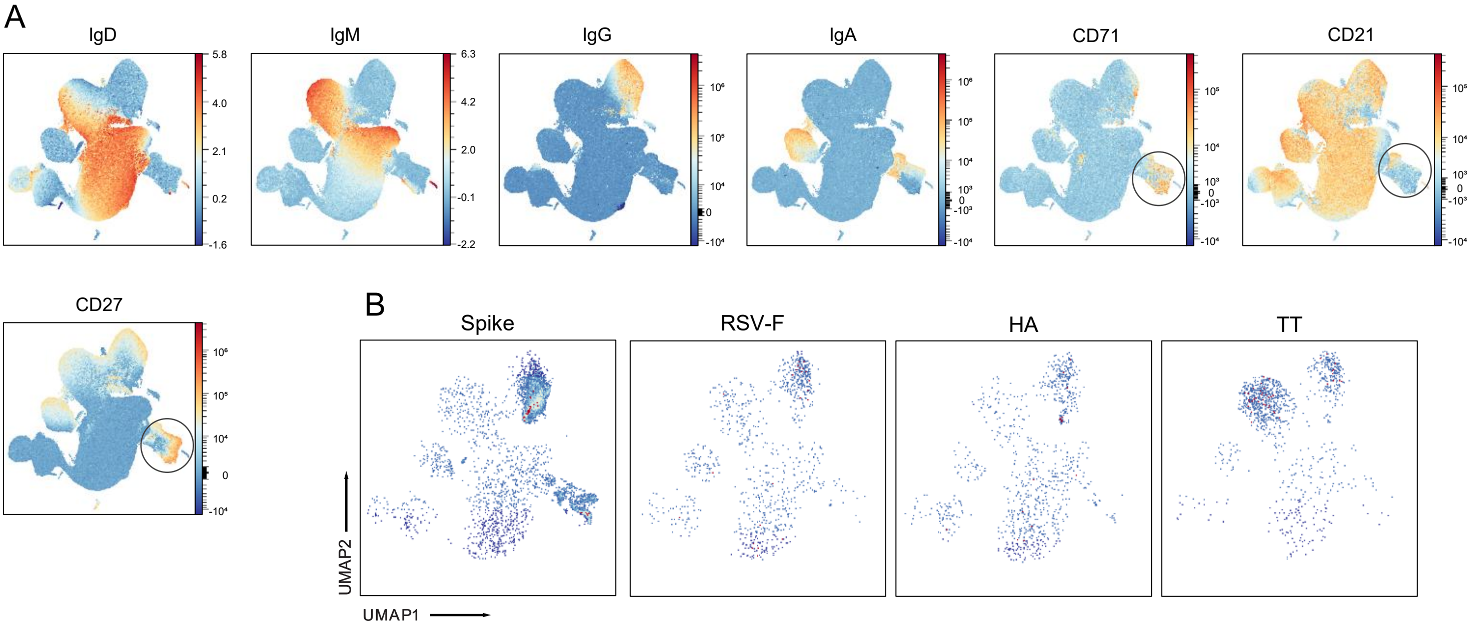

Supplementary fig.2. UMAP of total CD19$^+$ B cells.

a. UMAP representation of a subsample of 50.000 total CD19+ B cells performed only with the 18 healthy donors showing the expression of isotypes, CD71, CD21 and CD27. **b.** UMAP overlay of Spike, RSV-F, HA and TT-specific B cells included in the subsampling.

Supplementary Figure 3

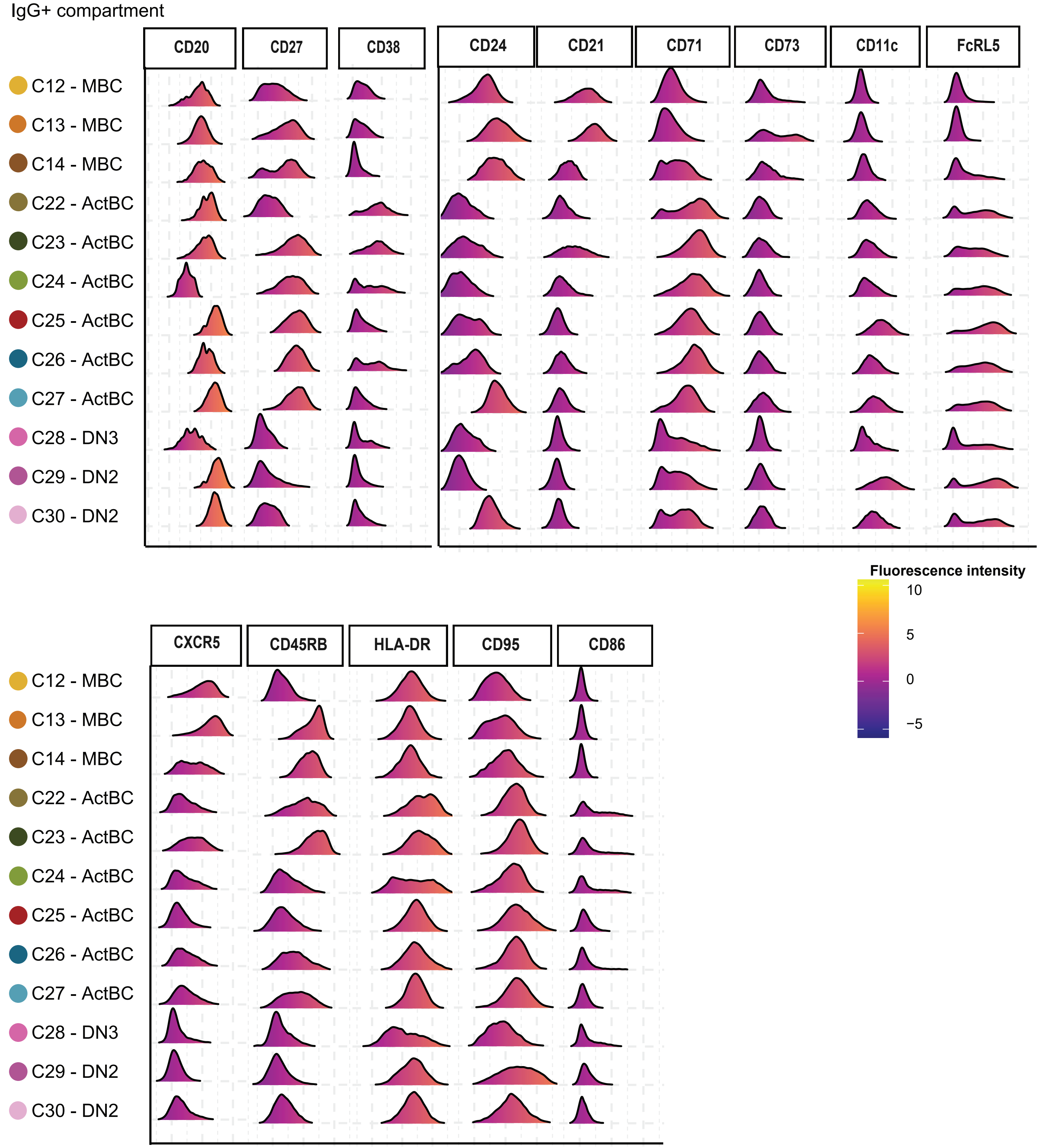

Supplementary fig. 3. Expression pattern of IgG^{+} ActBC, DN B cells and MBC clusters.
Analysis of cell surface expression by histogram representation of 14 relevant markers in IgG ActBC, DN B cell and MBC clusters.

Supplementary Figure 4

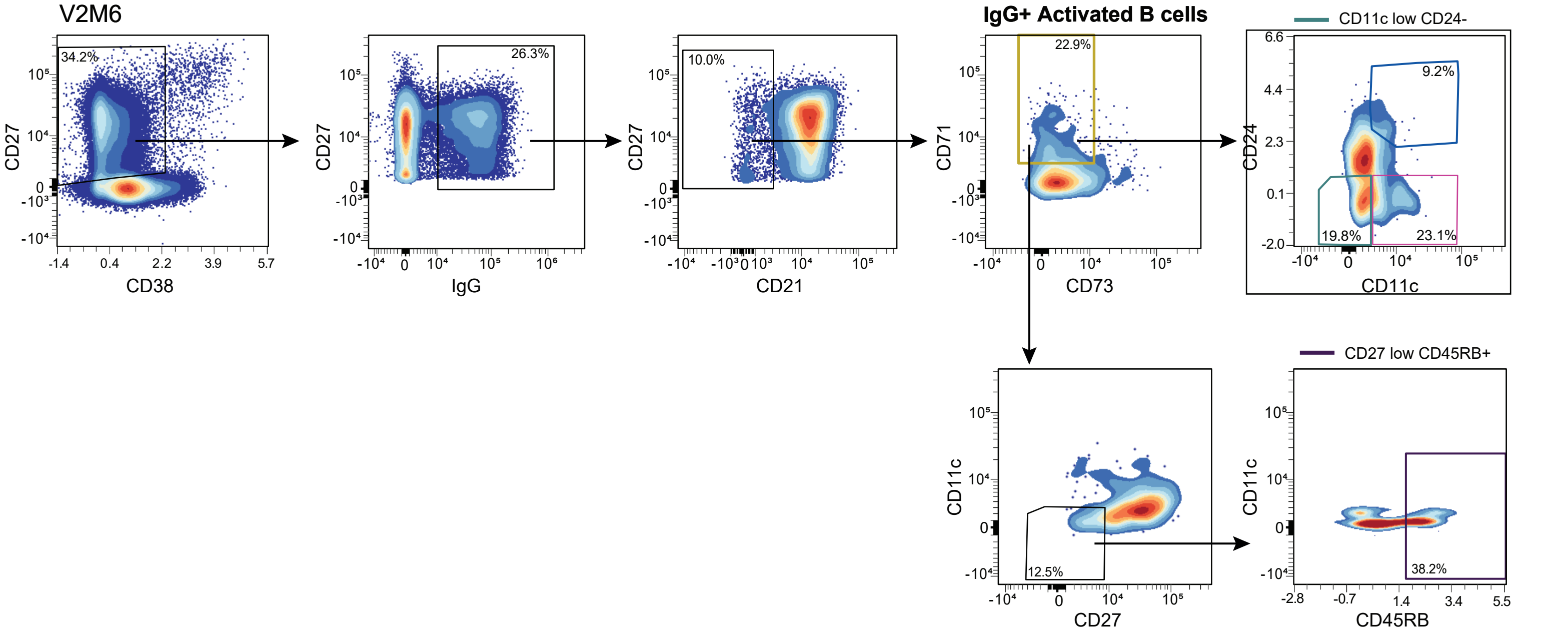

Supplementary fig. 4. Gating strategy of activated B cell subpopulations at V2M6.

Representative gating strategy to detect IgG+ ActBCs in total CD19+ B cells from healthy donor PBMCs at V2M6 and further CD24, CD11c, CD27 and CD45RB gating strategy.

Supplementary table 1. Panel design antigen probes.

Protein antigen	Fluorochrome-conjugated streptavidin	Concentration	Source	Catalog No
Wuhan SARS-CoV-2 Spike	Alexa Fluor® 647	0.5 ug/ul	BioLegend	405237
	Brilliant Violet™ 421	0.5 ug/ul		405225
Wuhan SARS-CoV-2 RBD	PE-Alexa Fluor® 610	1 ug/ul	Invitrogen	S20982
H1N1 HA	BUV615	0.1 ug/ul	BD	613013
	Brilliant Violet™ 421	0.5 ug/ul	BioLegend	405225
Nucleocapsid	Brilliant Blue™ 515	0.1 ug/ul	BD	564453
	Alexa Fluor® 647	0.5 ug/ul	BioLegend	405237
RSV F	BUV615	0.1 mg/mL	BD	613013
	Alexa Fluor® 647	0.5 ug/ul	BioLegend	405237
Tetanus toxoid	Brilliant Blue™ 515	0.1 ug/ul	BD	564453
	Brilliant Violet™ 421	0.5 ug/ul	BioLegend	405225

Supplementary table 2. B cell panel.

Antibody	Clone	Fluorochrome	Dilution[A]	Source	Catalog no
CD24	ML5	BUV395	1/80	BD	563818
Viability	-	Live/Dead Blue	1/1000	Thermo Fisher	L23105
HLA-DR	G46-6	BUV496	1/80	BD	749866
IgD	IA6-2	BUV563	1/160	BD	741394
CD126	M5	BUV661	1/20	BD	752527
CD138	MI15	BUV737	1/100	BD	612834
CD21	B-ly4	BUV805	1/160	BD	742008
CD20	2H7	cFluor V450	1/40	Cytek	SKU R7-20015
CD27	L128	BV480	1/20	BD	566188
CD3 (DUMP)	UCHT1	BV510	1/50	BioLegend	300448
CD4 (DUMP)	OKT4	BV510	1/50	BioLegend	317444
CD16 (DUMP)	3G8	BV510	1/50	BioLegend	302048
CD56 (DUMP)	HCD56	BV510	1/50	BioLegend	318340
CD19	HIB19	BV570	1/40	BioLegend	302236
CD11c	B-ly6	BV605	1/160	BD	563929
CXCR5	RF8B2	BV650	1/20	BD	740528
IgM	MHM-88	BV711	1/80	BioLegend	314540
CD71	M-A712	BV750	1/80	BD	747308
FcRL5	509F6	BV785	1/100	BD	749602
CD73	AD2	cFluor B532	1/80	Cytek	SKU R7-20017
CXCR3	1C6	BB700	1/25	BD	566532
CXCR4	12G5	PerCP-eFluor710	1/50	Thermo Fisher	46-9999-42
CD45RB	MEM-55	PE	1/80	BioLegend	310204
IgG	G18-145	PE-CF594	1/160	BD	562538
CD95	DX2	PE-Cy5	1/320	BioLegend	305610
CD30	BY88	PE-Cy7	1/12	BioLegend	333918
CD269	19F2	APC	1/25	BioLegend	357506
CD86	2331 (FUN-1)	APC-R700	1/320	BD	565149
IgA	IS11-8E10	APC-Vio770	1/320	Miltenyi Biotec	130-113-473
CD38	HIT2	APC-Fire810	1/160	BioLegend	303550

[A]Dilutions used were optimized in-house, these should be used as a guideline and optimized for individual laboratories

Supplementary table 3. Antigen-specific B cell cluster annotations.

Cluster	B cell subpopulation	B cell population	Isotype	Lineage markers	Dynamic markers	Other markers
1	IgD low CD73+ HLA-DR+ naive B cell	Naive B cell	IgD	CD27- CD21+	CD73++ HLA-DR++	CXCR5+
2	IgD/ IgM transitional B cell	Transitional B cell	IgD/ IgM	CD27- CD38+		CXCR5+
3	IgD/IgM CD73+ naïve B cell	Naïve B cell	IgD/ IgM	CD27- CD21+	CD24+ CD73+	CXCR5+ CXCR4+
4	IgD CD73+ naïve B cell	Naïve B cell	IgD	CD27- CD21+	CD24+ CD73+	CXCR5+ CXCR4+
5	IgM CD45RB+ MBC	IgM MBC	IgM	CD27+ CD21+	CD24+ CD45RB+	CXCR5+
6	IgM CD27low CD45RB+ MBC	IgM MBC	IgM	CD27low CD21+	CD24+ CD45RB+	CXCR5+
7	IgD CD73low CD45RB+ naïve B cell	CD45RB+ naïve B cell	IgD	CD27- CD21+	CD24+ CD73low CD45RB+	CXCR5+ CXCR4low
8	IgD/IgM CD73low CD45RB+ naïve B cell	CD45RB+ naïve B cell	IgD/ IgM	CD27- CD21+	CD24+ CD73low CD45RB+	CXCR5+ CXCR4low
9	IgA CD45RB- CD73- MBC	IgA MBC	IgA	CD27+ CD21+	CD24low CD73- CD45RB-	CXCR5+ CD11c-
10	IgA CD45RB+ CD73+ MBC	IgA MBC	IgA	CD27+ CD21+	CD24+ CD73+ CD45RB+	CXCR5+ CD11c-
11	Undefined	Not B cell	-	-	-	-
12	IgG CD21+ CD73- MBC	IgG MBC	IgG	CD27+ CD21+	CD24+ CD73- CD45RB-	CXCR5+
13	IgG CD21+ CD73+ CD45RB+ MBC	IgG MBC	IgG	CD27+ CD21+	CD24+ CD73+ CD45RB+	CXCR5+
14	IgG CD21- CD73- CD45RB+ MBC	IgG MBC	IgG	CD27+ CD21-	CD24+ CD73- CD45RB+	CXCR5-
15	IgA CD45RB+ CD24- Activated B cell	IgA ActBC	IgA	CD27+ CD21-	CD71+ CD24- CD45RB+	CD11c low CXCR3+ CXCR5-
16	IgA CD45RB- CD24+ CD11c+ Activated B cell	IgA ActBC	IgA	CD27+ CD21-	CD71+ CD24+ CD45RB-	CD11c+ CXCR3+ CXCR5-
17	IgA DN2	IgA DN2	IgA	CD27- CD21-	CD71- CD24- CD45RB-	CD11c++ CXCR3+ CXCR5-
18	IgD/IgM CD24+ MBC	IgD/IgM MBC	IgD/ IgM	CD27+ CD21-	CD71- CD24+ CD95+ CD45RB-	CXCR3+ CXCR5-
19	IgD/IgM CD21- CD24+ Activated B cell	IgD/IgM ActBC	IgD/ IgM	CD27- CD21-	CD71- CD24+ CD95low CD45RB-	CXCR3+

20	IgM CD21- CD71+ Activated B cell	IgD/IgM ActBC	IgM	CD27- CD21-	CD71+ CD24- CD95++ CD45RB-	CD11c low CXCR3+
21	IgD CD21- CD11c+ Activated B cell	IgD/IgM ActBC	IgD	CD27- CD21-	CD71- CD24- CD95 low CD45RB-	CD11c+
22	IgG CD27low CD45RB+ Activated B cell	IgG ActBC	IgG	CD27low CD21-	CD71+ CD24- CD95+ CD45RB+ HLA-DR+	CD11c low
23	IgG CD45RB+ Activated B cell	IgG ActBC	IgG	CD27+ CD21-	CD71+ CD24- CD95+ CD45RB+ HLA-DR+	CD11c low
24	IgG CD11c low CD24- CD20low Activated B cell	IgG ActBC	IgG	CD27+ CD21- CD20low	CD71+ CD24- CD95+ CD45RB- HLA-DR low	CD11c low
25	IgG CD11c+ CD24- Activated B cell	IgG ActBC	IgG	CD27+ CD21-	CD71+ CD24- CD95+ CD45RB- HLA-DR+	CD11c++
26	IgG CD11c low CD24- Activated B cell	IgG ActBC	IgG	CD27+ CD21-	CD71+ CD24- CD95+ CD45RB- HLA-DR+	CD11c low
27	IgG CD11c+ CD24+ Activated B cell	IgG ActBC	IgG	CD27+ CD21-	CD71+ CD24+ CD95+ CD45RB- HLA-DR+	CD11c++
28	IgG DN3	IgG DN3	IgG	CD27- CD21- CD20low	CD24- CD71- CD95+	CD11c-
29	IgG DN2	IgG DN2	IgG	CD27- CD21-	CD24- CD71- CD95+	CD11c++
30	IgG CD24+ DN2	IgG DN2	IgG	CD27- CD21-	CD24+ CD71- CD95+	CD11c+
31	IgA Plasma cell	IgA ASC	IgA	CD27+ CD38+ CD138+	CD86+ CD269+ CD45RB+	
32	IgA Plasmablast	IgA ASC	IgA	CD27+ CD38+ CD138-	CD86+ CD269+ CD45RB+	
33	IgM Plasmablast	IgM ASC	IgM	CD27+ CD38+ CD138-	CD86+ CD269+ CD45RB+	
34	IgG Plasmablast	IgG ASC	IgG	CD27+ CD38+ CD138-	CD86+ CD269+ CD45RB+	
35	IgG Plasma cell	IgG ASC	IgG	CD27+ CD38+ CD138+	CD86+ CD269+ CD45RB+	

36	IgG CD20+ CXCR4+ CD30+ Early Plasmablast	IgG ASC	IgG	CD20+ CD27low CD38+ CD138-	CD86+ CD269+ CD45RB low CD30+	CXCR4+
37	IgG Early Plasmablast	IgG ASC	IgG	CD27low CD38+ CD138-	CD86+ CD269+ CD45RB low	
38	IgA Early Plasmablast	IgA ASC	IgA	CD27low CD38+ CD138-	CD86+ CD269+ CD45RB low	

Abbreviations: ASC, antibody secreting cell**;** ActBC, activated B cell**;** DN, double negative B cell; MBC, memory B cell
